# Supplementary material for: Crohn’s Disease Susceptibility and Onset Are Strongly Related to Three NOD2 Gene Haplotypes
Source: J Clin Med. 2021 Aug 24;10(17):3777. doi: 10.3390/jcm10173777 (PMC8432186; doi:10.3390/jcm10173777)
Supplement: Supplementary file 1 [file jcm-10-03777-s001.zip › Sup_Table S1.pdf]

Table S1. Primers used for genotyping the *NOD2* gene with high resolution melting analysis (HRMA) and Sanger sequencing and pyrosequencing (PyroSeq). Primers marked with * were biotinylated. F – forward primer, R – reverse primer, S – sequencing primer, Tm – primers annealing temperature.

**SNP**	**Method**	**Primer Sequence (5'-3')**	**PCR product (bp)**	**Tm (°C)**
rs2066842<br>c.802C>T<br>p.P268S	HRMA	F: GGAGGTCTGGGCAGATGTG<br>R: ACAGTGTCCGCATCGTCA	114	56
	PyroSeq	F: GGAGGTCTGGGCAGATGTG*<br>R: ACAGTGTCCGCATCGTCA<br>S: GGGCTCTCTGCGGG	144	60
rs2066844<br>c.2104C>T<br>p.R702W	HRMA	F: GAGCCGCACAACCTTCAGAT<br>R: GCGGGATGGAGTGGAAGT	172	64
	PyroSeq	F: GAGCCGCACAACCTTCAGAT*<br>R: GCGGGATGGAGTGGAAGT<br>S:GGCACAGGCCTGGCG	172	60
rs2066845<br>c.2722G>C<br>p.G908R	HRMA	F: GACTCTTTGGCCTTTTCAGATT<br>R: CCAATGGTCTTTTTTCCTTACTCC	242	58
	PyroSeq	F: GACTCTTTTGGCCTTTTCAGATT*<br>R: CCAATGGTCTTTTTTCCTTACTCC<br>S: TCGTCACCCACTCTGT	242	60
rs5743289<br>c.2798+158C>T<br>IVS8+158	HRMA	F: TGCAGTTTTCTTGGGGAGA<br>R: TGTACCTGATCCAGCCCAA	231	58
rs5743293<br>c.3020insC<br>p.1007fs	HRMA	F: GGGACAGGTGGGCTTCAGTA<br>R: CCATTCCTCTCTCCCGTCAC	279	55
	PyroSeq	F: ACCTACCTAGGGGCAGAAGC<br>R: CAGACTTCCAGGATGGTGTCA*<br>S: CCCTCCTGCAGGCCC	65	60

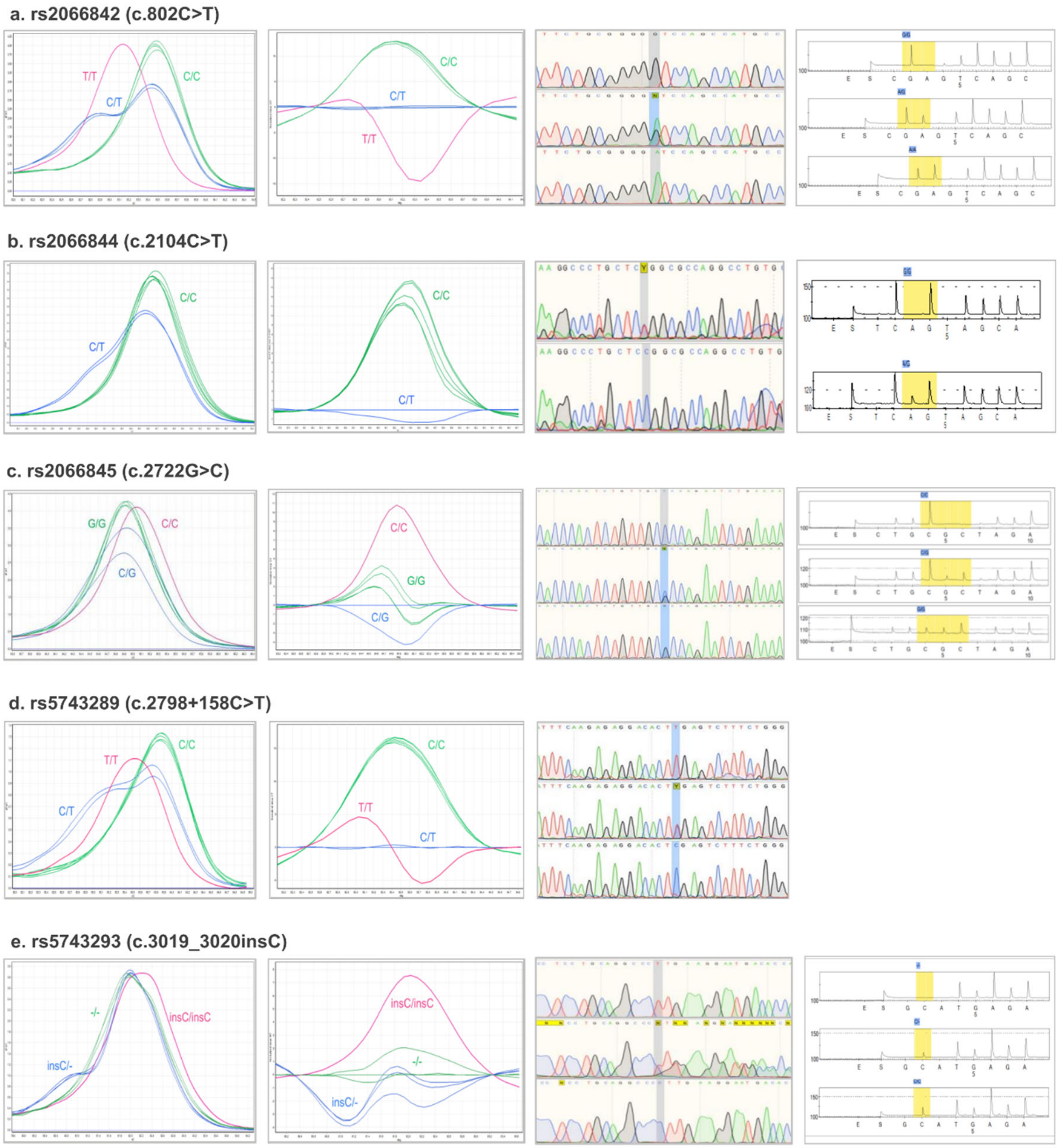

Supplementary Figure S1. Genotyping results for *NOD2* gene variants: a.) c.802C>T (p.P268S, rs2066842), b.) c.2104C>T (p.R702W, rs2066844), c.) c.2722G>C (p.G908R, rs2066845), d.) c.2798+158C>T, rs5743289, e.) c.3020insC (p.1007fs rs5743293) obtained using HRMA (melting curves and differential graphs), pyrosequencing and Sanger sequencing. Rs5743289 *locus* was genotyped only with HRMA and Sanger sequencing. In the case of a.) c.802C>T variant, sequencing primers (Sanger and pyrosequencing) were reverse; in case of b.) c.2104C>T variant Sanger sequencing primer was forward and pyrosequencing primer was reverse, c.), d.) and e.) – sequencing primers were forward.
